# Photodegradation of Microplastics through Nanomaterials: Insights into Photocatalysts Modification and Detailed Mechanisms

**DOI:** 10.3390/ma17112755

**Published:** 2024-06-05

**Authors:** Yiting Xiao, Yang Tian, Wenbo Xu, Jun Zhu

**Affiliations:** 1 Department of Biological Engineering, University of Arkansas, Fayetteville, AR 72701, USA; 2 Program of Material Science and Engineering, University of Arkansas, Fayetteville, AR 72701, USA; yangtian@uark.edu; 3 Department of Biomedical Engineering, University of Arkansas, Fayetteville, AR 72701, USA; wenboxu@uark.edu

**Keywords:** microplastics, photocatalysis, element doping, heterojunction, plasmonic photocatalysts, mechanism

## Abstract

Microplastics (MPs) pose a profound environmental challenge, impacting ecosystems and human health through mechanisms such as bioaccumulation and ecosystem contamination. While traditional water treatment methods can partially remove microplastics, their limitations highlight the need for innovative green approaches like photodegradation to ensure more effective and sustainable removal. This review explores the potential of nanomaterial-enhanced photocatalysts in addressing this issue. Utilizing their unique properties like large surface area and tunable bandgap, nanomaterials significantly improve degradation efficiency. Different strategies for photocatalyst modification to improve photocatalytic performance are thoroughly summarized, with a particular emphasis on element doping and heterojunction construction. Furthermore, this review thoroughly summarizes the possible fundamental mechanisms driving the photodegradation of microplastics facilitated by nanomaterials, with a focus on processes like free radical formation and singlet oxygen oxidation. This review not only synthesizes critical findings from existing studies but also identifies gaps in the current research landscape, suggesting that further development of these photocatalytic techniques could lead to substantial advancements in environmental remediation practices. By delineating these novel approaches and their mechanisms, this work underscores the significant environmental implications and contributes to the ongoing development of sustainable solutions to mitigate microplastic pollution.

## 1. Introduction

Over the past six decades, plastic production has increased substantially due to its low cost, durability, and versatility. Our world produced 460 million tons of plastic in 2019, which is 230-fold of what was produced in 1950 [1]. This massive increase in plastic production has resulted in significant environmental challenges, including pollution of land and water bodies [2], harm to wildlife [3,4], and contribution to climate change [5]. Plastics exhibit extreme durability in marine environments, often persisting for several hundred years before degrading completely. For instance, studies have shown that polyethylene (PE) can take up to 292 years to fully degrade in the deep sea due to the unique conditions of low temperature, absence of UV light, and depleted oxygen levels [6]. These degradation processes, driven primarily by microbial activity, highlight the long-term environmental persistence and potential for the widespread distribution of microplastics in marine ecosystems. This slow degradation exacerbates the risk of bioaccumulation in marine organisms, further escalating the threat to both human health and marine biodiversity. Microplastics (MPs) are small plastic particles that are less than 5 mm (0.20 in) in size. MPs can be classified based on their origin as either primary or secondary. Primary MPs consist of plastic items that are already smaller than 5 mm and originate from products such as exfoliating beads in cleaning products and fibers from clothing, whereas secondary MPs are formed from the fragmentation of larger plastic debris. Microplastics are prevalent across various aquatic environments, posing significant ecological challenges. In domestic wastewater, despite treatments removing up to 99.9% of microplastics, facilities still release millions of particles daily [7,8]. Rivers like the Austrian Danube show high microplastic concentrations, with abundance reaching 316.8 ± 4664.6 items per 1000 m^3^, surpassing the abundance of drifting larval fish [9]. In lakes, studies in the Laurentian Great Lakes detected up to 1.5 microplastics per liter in surface waters and up to 27,830 particles per kg of dry mass in Benthic [8]. These figures illustrate the urgent need for targeted environmental management strategies to mitigate microplastic pollution. When aquatic creatures ingest MPs, these materials are hard to break down and can inflict physical harm, such as internal abrasions and blockages, potentially resulting in a slow death [10,11]. Additionally, when plastic items age and fragment, many additives in plastic products slowly desorb into the environment, such as polybrominated diphenyl ethers (PBDEs), phthalates, nonylphenols, and bisphenol A (BPA) [12]. The consumption of toxic MP by-products can lead to various detrimental consequences, including carcinogenesis, endocrine disruption, impaired reproduction, and malnutrition [10,11,13]. Furthermore, due to their hydrophobicity and large specific surface area, MPs can absorb pollutants in the environment, which affects the migration, transformation, and bioavailability of pollutants [14,15]. What is worse, because of the intricate dynamics of the food chain, plastic infiltration has profound effects on marine life, agricultural produce, fauna, and humans [16,17]. According to the study conducted by Leslie et al. [18], at least five types of MPs, including polypropylene (PP), polystyrene (PS), polymethyl methacrylate (PMMA), polyethylene (PE), and polyethylene terephthalate (PET), were found in the bloodstreams of approximately 80% of survey participants. Additionally, human feces and the placenta have also been found to contain MPs, which indicates that MPs are entering our bodies from everyday items like beverage bottles and food packaging. Humankind and the delicate balance of the encompassing ecosystem are threatened by this insidious contamination, which necessitates immediate attention and action to mitigate its deleterious consequences. Although some countries, including the US with the Microbead-Free Water Act [19], have prohibited the use of microbeads in personal care products, this only addresses a small portion of the overall MPs that are released to the environment and only accounts for 2% of the primary MPs entering the global marine ecosystem [20]. It is, therefore, essential to find effective ways to remove MPs to ensure aquatic ecological safety and human health.

Microplastic (MP) degradation can be achieved by using a variety of physical, chemical, and biological methods, including coagulation [21], hydrolysis [22], ozonation [23], chlorination [24], membrane technology [25], green algae [26], and active sludges [27]. However, each method presents its own set of limitations and tradeoffs in terms of demands on energy, efficiency, limitation of applicability to specific types of MPs, the challenge of fine control, and the potential for environmental impact. A summary of different treatment methods for treating MPs is shown in Table 1. Photodegradation is emerging as one of the promising and environmentally friendly approaches to MP decomposition that may be able to address some of the challenges. By utilizing light energy, photodegradation breaks down MP chemical bonds and transforms them into smaller, less harmful compounds. Since photodegradation does not require additional chemicals and does not generate significant by-products or waste, it can be an efficient, cost-effective, and environmentally friendly alternative to other methods. Unlike the widely studied water splitting, which requires photocatalysts to have a bandgap of at least 1.23 eV [28], the photocatalytic process on MP has a much lower energy requirement by utilizing a wide spectrum of solar energy, including UV, visible light, and even near-infrared light, suggesting its immense potential for addressing the pervasive problem of MP pollution. Photocatalytic processes lead to an increase in not only the carbonyl index of residues, but also the brittleness, wrinkles, cracks, and cavities on the surface of MPs [29], and convert them to CO_2_ and H_2_O eventually. This green technology offers a sustainable solution to not only reduce the accumulation of plastic waste but also minimize the associated risks posed to living organisms and the environment. As research and innovation continue to advance in this area, photodegradation could potentially become a key strategy in the global effort to manage and remediate plastic pollution.

The success of large-scale MP degradation rests heavily on amplifying this efficiency. While myriad factors play a role in the degradation process, everything essentially revolves around the potency of photocatalysts themselves. To thoroughly investigate this area, we conducted a focused literature review, searching databases like Web of Science and Google Scholar for recent studies (past 10 years) on modifications of photocatalysts and their effectiveness in microplastic degradation. However, a significant portion of contemporary research only focused on traditional nanomaterials such as TiO_2_ or ZnO, celebrated for their expansive bandgap, durability, and cost-effectiveness. However, these nanomaterials are confronted with inherent challenges, most prominently the accelerated recombination of electron-hole pairs and suboptimal performance within visible light spectrums, thus leading to a low efficiency in the degradation of the MPs. Modifying photocatalysts to enhance their photodegradation efficiency for MPs is not only desirable but also essential. To effectively address this issue, it is essential to expand the scope of research beyond single metal oxides to include a diverse array of modified photocatalysts, including plasmonic photocatalysts [39], doped photocatalysts [40], composite photocatalysts [41], and oxygen deficient photocatalysts [42], etc. In addition, the photocatalytic performance of semiconductors, which play a crucial role in the photodegradation process, is also determined by factors such as composition, surface state, crystallinity, band structure, morphology, and interfacial properties between the components in composite photocatalysts [43]. Given the pressing environmental threat posed by MPs and the limitations of current solutions, this review uniquely synthesizes recent advancements in photocatalyst modification and the underlying mechanistic actions of MP photocatalysis, addressing a significant gap in comprehensively understanding the photocatalyst composition and structural properties needed for effective MP degradation. It offers detailed discussions on novel approaches that could overcome the limitations of current photocatalytic strategies, directly contributing to the development of more sustainable and efficient environmental remediation technologies. 

## 2. Enhancement of Photocatalytic Efficiency

The semiconductor photocatalyst is a material that is capable of absorbing light and generating electron-hole pairs, which can then drive a variety of chemical reactions, such as splitting of water [44], reduction of CO_2_ [45], or degradation of pollutant emissions [46]. In general, photocatalysts can be divided into two categories. The first category is semiconductor photocatalysts, which include metal-containing semiconductors (such as oxides, nitrides, sulfides, and oxynitrides) and other non-metal semiconductors (such as graphitic carbon nitride (g-C3N4), graphene oxide (GO) [47], and carbon quantum dots (CQDs)) [48]. The second category is plasmonic photocatalysts, which are composed of metal nanoparticles (NPs), such as copper, gold, and silver, that can exhibit strong localized surface plasmon resonance (LSPR) properties under visible light [49]. These plasmonic photocatalysts can use the unique properties of noble metals to enhance light absorption and thereby increase photocatalytic activity. 

The band structure of photocatalytic materials, including bandgap width, band position, and band bending, plays a pivotal role in determining the efficiency of photocatalysis. By effectively controlling these parameters, one can significantly improve the photogenerated charge carrier migration and, thus, the catalytic activity. Band structure can be tuned through various methods, while the most used methods in the degradation of microplastics are (1) doping, where intentional impurities are introduced into a semiconductor to modulate its electronic properties, and (2) the creation of heterostructures, which involves the integration of two or more different semiconductor materials to form a composite with superior properties.

### 2.1. Element Doping

**Figure 1:**
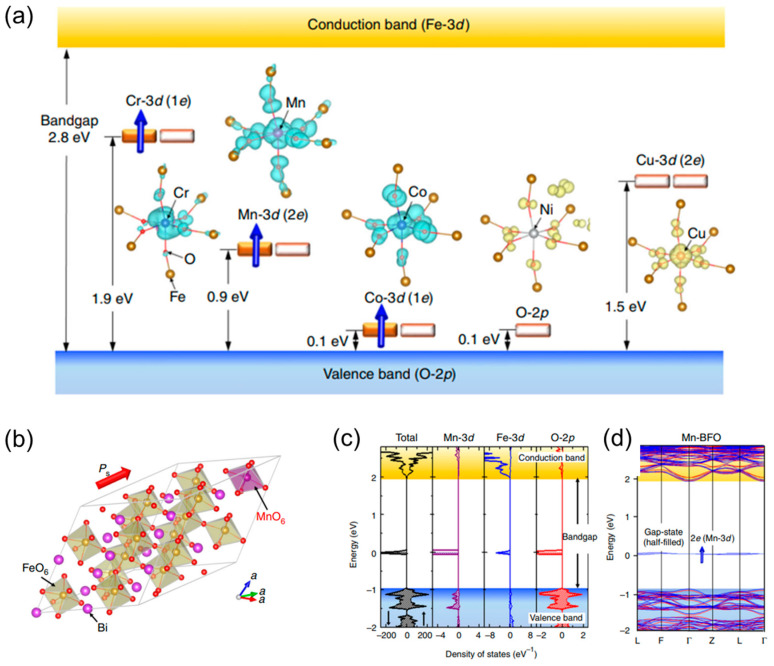
(**a**) Wave functions and schematic electronic structures of transition metal (TM = Cr, Mn, Co, Ni, and Cu)-doped BFO obtained from DFT calculations. The blue arrows designate spin-polarized electrons in the gap states. Crystal and electronic structures of Mn-doped BFO. (**b**) Optimized crystal structure of Mn-doped BFO [Bi_16_(Fe_15_Mn)O_48_] in rhombohedral R3 symmetry, (**c**) total density of states (DOS) and partial DOS (PDOS) of Mn-3d, Fe-3d and O-2p and (**d**) electronic band structure. The majority-spin and minority-spin states are depicted by red and blue lines, respectively, with the blue arrow highlighting a spin-polarized electron in the gap state, demonstrating that Mn generates the half-filled gap (2e) state [54] Copyright © 2017 Springer Nature Limited.

Doped photocatalysis has emerged as a prominent technique to optimize the photocatalytic abilities of nanostructures. Doping introduces defects into the ideal crystal lattice of the native semiconductor and modifies the electronic structure of the photocatalysts in order to improve their activity [50,51]. This process not only helps in retarding the rapid charge recombination but also enables the absorption of visible light. This is because it enhances the interfacial charge transfer by trapping either the valence band (VB) holes or conduction band (CB) electrons within these defective sites [52]. In addition, doping also increases the sub-bandgap irradiation, as shown in Figure 1, which allows electronic transitions from the defect states to the CB or from the VB to the defect states [53,54]. 

The variety of dopants mainly falls into two categories: metal ions and non-metal ions. Typically, metals can facilitate electron transfer and reduce the band gap energy level due to their inherent potential to transfer electrons, so they are more favored than non-metal dopants in modifying photocatalysts [53,55]. When metal (cationic) dopants, either transition or noble, are introduced into the photocatalyst matrix, these dopants play a pivotal role in adjusting the semiconductor’s bandgap. Doping with different metal ions possessing varied valence states can alter the material’s light absorption range, vastly expanding the semiconductor’s visible absorption range. For example, researchers like Tripathi et al. [56] demonstrated that Sn and Mn doping in TiO_2_ nanoparticles, prepared via the sol-gel method, led to a red shift with the bandgap energy decreasing from 3.24 eV to as low as 2.21 eV for Sn and 2.56 eV for Mn. Yao et al. [57] found that the synthesized Fe(III) porphyrin-conjugated TiO_2_ has a strong response in the 500–800 nm range, whereas bare TiO_2_ has no absorption above 400 nm. Essentially, when metal ions are infused into the photocatalyst, it causes the genesis of a new energy level, which then hampers the recombination of electron/hole pairs. This phenomenon plays a pivotal role in enhancing photocatalytic activity under visible light. 

**Figure 2:**
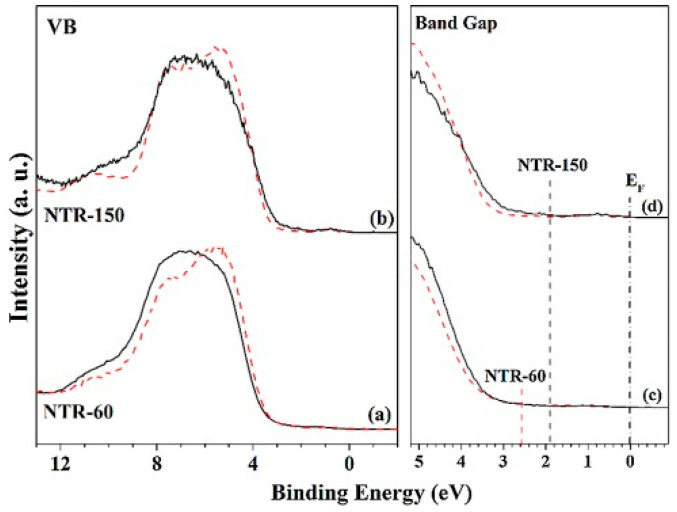
(**a**,**b**) Valence spectra and (**c**,**d**) band gap measurements of (**a**,**c**) NTR-60 and (**b**,**d**) NTR-150. (Black lines correspond to experimental data, while red dashed lines represent non-doped TR-60 and TR-150) [59] © 2016 Elsevier B.V. All rights reserved.

In contrast to metal ions, non-metal ions, such as B, C, N, F, S, and P, generally do not act as recombination centers for charge carriers. Instead, they elevate the semiconductor material’s band position, narrowing the bandgap or introducing a new energy level to trap charge carriers, thereby improving the migration efficiency of photogenerated charge carriers. Except for boosting the visible light response, Viswanathan et al. [58] reported that nitrogen (N) doping also modified several other properties of TiO_2_, such as its hardness, refraction index, and electrical conductivity. A more specific exploration into the effects of N-doping can be seen in the work of Hwang et al. [59], as shown in Figure 2. They synthesized N-doped TiO_2_ nanorods (NTR) and revealed a significant reduction in their bandgap from the typical 3.0 eV, as found in pristine rutile TiO_2_, down to 1.94 eV. Detailed spectral analysis attributed this reduction to Ti^3+^ and oxygen vacancy defect states. Furthermore, they discovered that long N-doped nanorods (NTR-150) samples possess a narrower bandgap than the short ones (NTR-60), which is possibly due to their richer defect structures and higher N-doping levels. This observation underscores the significant influence of both N-doping and defect structures in determining the bandgap of these N-doped TiO_2_ nanorods. Consequently, this results in a superior photo-oxidation activity for NTR-150 when compared to NTR-60.

This improvement in photocatalysis efficiency has also been found during the degradation of MPs by N-doped nanomaterials. For example, Ariza-Tarazona et al. [60] demonstrated that the C, N-TiO_2_ that they synthesized could degrade 72% of high-density polyethylene (HDPE) in 50 h. However, due to the high persistence of MPs, hydrothermal pretreatment was also used as a pretreatment method for photodegradation. For example, Kang et al. [61] demonstrated the possibility of using magnetic N-doped nanocarbon springs that were capable of activating peroxymonosulfate to generate reactive radicals and decompose MPs under hydrothermal conditions. In addition, the nano-flower shaped N doped-TiO_2_ catalyst (Pt@N-TiO_2_-1.5%) synthesized by Zhou et al. [62], after undergoing hydrothermal pretreatment, demonstrated an ability to degrade polyethylene terephthalate that was eight times more effective than the untreated sample.

### 2.2. Heterojunction Construction

**Figure 3:**
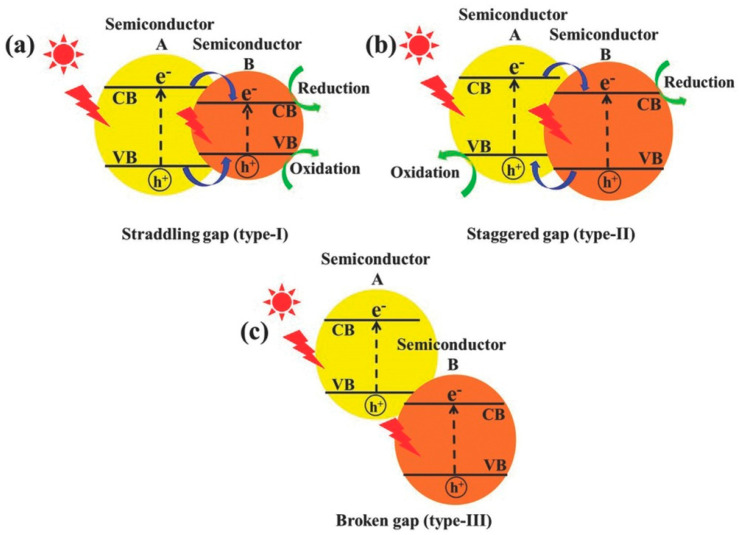
Schematic illustration of the three different types of separation of electron–hole pairs in the case of conventional light-responsive heterojunction photocatalysts: (**a**) type–I, (**b**) type–II, and (**c**) type–III heterojunctions. The blue arrows representing the movement of holes or electrons, while the green arrows representing the chemical reactions [64] © 2017 WILEY-VCH Verlag GmbH & Co. KGaA, Weinheim.

A heterojunction refers to the interface between two or more distinct semiconductors having different band structures, leading to specific band alignments [63]. Traditionally, there are three primary types of traditional heterojunction photocatalysts: type-I (straddling gap), type-II (staggered gap), and type-III (broken gap), as illustrated in Figure 3. In a type-I heterojunction, the conduction band (CB) and the valence band (VB) of one semiconductor are positioned above and below the respective bands of the second semiconductor. This alignment causes both electrons and holes to accumulate on the same semiconductor under light exposure, resulting in ineffective electron-hole separation and reduced redox ability. 

In contrast, the type-II heterojunction exhibits an alignment where both the CB and VB of one semiconductor are higher than those of the other. Upon light irradiation, this configuration promotes electron migration to one semiconductor and hole migration to the other, achieving efficient spatial separation of electron-hole pairs. However, the redox ability of this heterojunction is also reduced due to distinct redox reactions occurring on separate semiconductors. The type-III heterojunction resembles the type-II alignment but with an extremely staggered gap, causing the bandgaps not to overlap and thus making electron-hole migration and separation between the semiconductors impossible. Among these, the type-II heterojunction stands out as the most promising for enhancing photocatalytic activity. The appropriate overlap of energy bands ensures that the electrons and holes accumulate in different materials, making them more conducive to the spatial separation of the electron-hole pairs, thus allowing their participation in ensuing redox reactions. Over the years, there has been significant emphasis on developing type-II heterojunction photocatalysts, such as TiO_2_/g-C_3_N_4_, BiVO_4_/WO_3_, and g-C_3_N_4_–BiPO_4_, which have demonstrated commendable electron-hole separation efficiency, broad light-absorption capability, and rapid mass transfer.

The type-II heterojunction, while theoretically promising for spatial separation of photogenerated electron-hole pairs upon illumination, carries certain intrinsic drawbacks that hamper its practical application. Thermodynamically, the efficiency of separating these photogenerated electron-hole pairs comes at the expense of reducing the redox potential of both semiconductor photocatalysts involved. For specific reactions that necessitate a certain redox potential, this reduction may hinder them. From a kinetics perspective, the presence of electrostatic interactions can inhibit interfacial charge transfer, as photogenerated charges in one catalyst suppress those in another due to mutual repulsion. 

To further improve the photocatalytic efficiency, p–n heterojunction photocatalyst emerges as one promising alternative. The internal electric field of the p–n heterojunction can serve as a driving force to promote the separation of electrons and holes. To be more specific, holes in the p-region would diffuse into the n-region until the Fermi energy levels are equated, leaving a net negative charge in the p-region. Conversely, the n-region accumulates a net positive charge, forming a built-in electric field from the n-region to the p-region. Under light exposure, photo-generated electrons and holes move in opposite directions due to this internal field, thus achieving their separation. Moreover, Qin et al. [65] synthesized an enhanced Ag_2_O/Fe-MOF p-n heterojunction photocatalyst, which excelled in converting microplastics such as PEG, PE, and PET while also producing H_2_. Notably, it facilitated the selective transformation of MPs into value-added products, presenting a promising approach to environmental remediation through microplastic upcycling and hydrogen generation.

**Figure 4:**
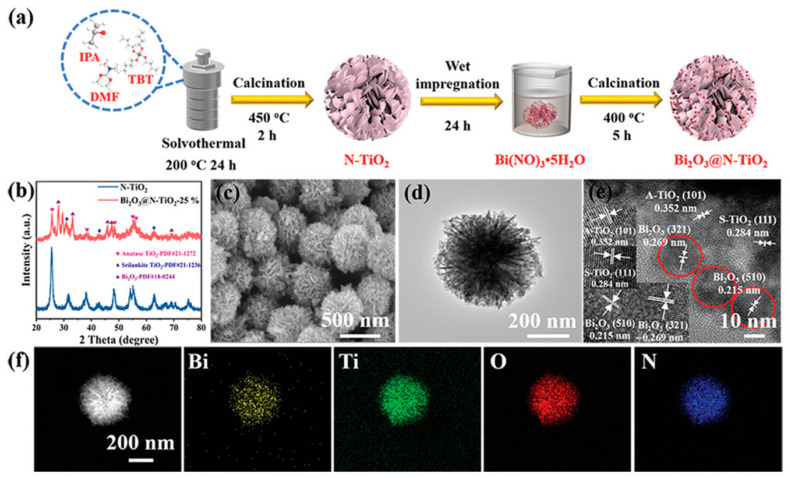
(**a**) Schematic diagram of the preparation process of the Bi_2_O_3_@N-TiO_2_. (**b**) X-ray diffractometer (XRD) patterns of N-TiO_2_ and Bi_2_O_3_@N-TiO_2_-25%. (**c**) Scanning electron microscope (SEM) image, (**d**) transmission electron microscopy (TEM) image, (**e**) HRTEM image of Bi_2_O_3_@N-TiO_2_ with and Bi_2_O_3_ nanoparticles highlighted in red circles, and (**f**) EDS elemental mappings of Bi_2_O_3_@N-TiO_2_-25%. The internal picture of (**e**) is the enlarged image of the lattice spacing of N-TiO_2_ and Bi_2_O_3_ [68] © 2022 Wiley-VCH GmbH.

The Z-scheme heterojunction, inspired by the natural photosynthetic charge transfer process in plants [66], serves as another promising alternative to the limitations of type-II heterojunctions. First introduced by Bard et al. [67] in 1979, the concept aimed to simulate natural photosynthesis and enhance the redox potential of photocatalysts. A typical Z-scheme photocatalytic system consists of two staggered semiconductor photocatalysts, PS I and PS II, connected by an acceptor/donor (A/D) pair, thereby allowing the spatial separation of electron-hole pairs. During the photocatalytic process, electrons migrate from the conduction band (CB) of PS II to the valence band (VB) of PS I through this A/D pair, ensuring optimal redox ability. This system achieves both spatial separation of the redox sites and ensures that photocatalysts maintain appropriate valence band positions, preserving a strong redox reaction ability. For example, Zhou et al. [68] have successfully developed a Z-scheme Bi_2_O_3_@N-TiO_2_ heterojunction utilizing a combination of solvothermal and wet-impregnation techniques in Figure 4. This advanced heterojunction showcases its potential by degrading approximately 10.23 ± 1.91 wt% of PET fiber-based microplastic (FMP)—a predominant form of FMP pollution in the environment—under alkaline conditions (pH = 9).

Although these architectures seek to address some of the limitations of the type-II heterojunction and improve charge separation efficiency, the introduction of an A/D pair, essential for facilitating charge transfer between the two semiconductors, raises new challenges. The Z-scheme is confined to liquid-phase reactions due to the need for the A/D pair to achieve sufficient migration rates [69]. Potential side reactions, influenced by a larger potential difference, can disrupt the charge transfer process [70]. Furthermore, the redox pairs might exhibit color, as seen in ferrous/ferric pairs, which could interfere with light absorption by the photocatalyst [71]. The system’s functionality also hinges on maintaining specific pH conditions. While the Z-scheme offers a unique approach to enhancing electron-hole separation, it, too, comes with its set of challenges that constrain its broader applicability in photocatalysis.

This has spurred innovations of the S-scheme heterojunctions, introduced by Fu et al. [72] in 2019 as the “step-scheme heterojunction”, which aims to combine efficient charge separation with potent photo-redox capabilities. Distinctly, the S-scheme heterojunction comprises a reducing photocatalyst (RP) with a smaller work function and a higher Fermi level, juxtaposed with an oxidizing photocatalyst (OP) possessing a larger work function and a lower Fermi level. When structured in this staggered fashion, they can effectively facilitate the separation of electron-hole pairs that boast strong oxidation-reduction capabilities.

**Figure 5:**
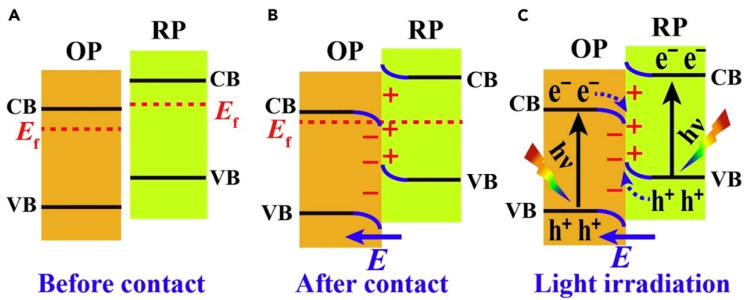
Charge-Transfer Processes in an S-Scheme Heterojunction (**A**–**C**) schematic illustration of heterojunction with staggered band configuration: (**A**) before contact, (**B**) after contact, (**C**) photogenerated charge carrier transfer process in S-scheme mode. The blue arrows representing the directions of the internal electric field [75] © 2020 Elsevier Inc.

Visually, the structure of the S-scheme heterojunction bears a resemblance to a type-II heterojunction. However, there is a key distinction in their functional mechanisms. As shown in Figure 5, In a typical type-II heterojunction, photo-induced electrons and holes tend to accumulate on the conductive and valence bands of the RP and OP, respectively, which can diminish their redox potential. In contrast, the S-scheme maintains effective electrons and holes while allowing non-essential photogenerated carriers to recombine. This step-like electron transfer mechanism is the defining characteristic of the S-scheme heterojunction, thus its name.

The charge transfer process in an S-scheme heterojunction is underlined by three pivotal factors: an intrinsic electric field, band bending, and electrostatic interactions [73,74]. Due to RP’s smaller work function and elevated Fermi level, when OP and RP come into contact, electrons spontaneously diffuse from RP to OP, establishing an electron depletion layer and an accumulation layer. This makes OP negatively charged and RP positively charged, creating an intrinsic electric field. This field facilitates the transfer of photogenerated electrons from OP to RP. Furthermore, when both semiconductors touch, their Fermi levels align at a common energy level, causing band bending between OP and RP, prompting the recombination of conductive electrons in OP with valence holes in RP. The electrostatic interactions at the interface of the two semiconductors also encourage different electrons and holes to recombine. This intricate interplay ensures spatial separation of the photogenerated electron-hole pairs while retaining robust oxidation–reduction capabilities.

### 2.3. Other Improvement Strategies

#### 2.3.1. Sensitizers

Sensitizers are widely used in photochemistry, particularly when direct light absorption is difficult due to low absorption coefficients or competing processes such as fluorescence [76]. A lower level of electronic excitation energy is transferred to the substrate after the light has been absorbed. This also results in an increased degree of chemical selectivity. Photosensitization can extend the light absorption range, enhance photon harvesting efficiency, provide additional excited electron pairs from a dye, and accelerate charge transfer, thus leading to higher photoelectric conversion efficiency [77]. For example, Zhao et al. demonstrated that copper phthalocyanine (CuPc) modifications enhanced TiO_2_ charge separation efficiency, resulting in a threefold increase in PE photodegradation efficiency [78]. 

#### 2.3.2. Defect Engineering

**Figure 6:**
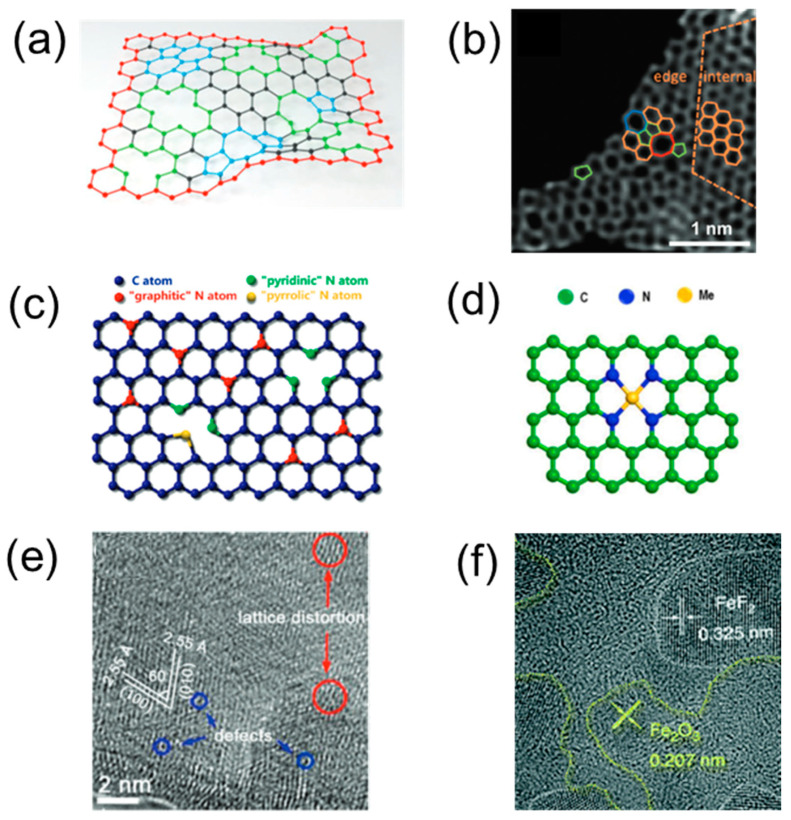
(**a**) Schematic illustration of point defects and edges in graphene materials. Red atoms denote edges, green atoms denote vacancies, and blue atoms denote topological defects. Reprinted with permission from [83] Copyright 2018, John Wiley and Sons. (**b**) HAADF image of graphene possessing defects. Hexagons, pentagons, heptagons, and octagons were labeled in orange, green, blue, and red, respectively. Reprinted with permission from [84]. Copyright 2016, John Wiley and Sons. (**c**) Schematic representation of N-doped graphene. Reprinted with permission from [85]. Copyright 2009, American Chemical Society. (**d**) General molecular structures of the M–Nx–C material. Reprinted with permission from [86]. Copyright 2017, American Chemical Society. (**e**) HRTEM images of δ-FeOOH nanosheets. Reprinted with permission from [87]. Copyright 2018, John Wiley and Sons. (**f**) HRTEM images of FeF_2_–Fe_2_O_3_ hybrid. Reprinted with permission from [88]. Copyright 2018, Springer Nature.

Defect engineering of nanomaterials also provides an effective way to enhance photocatalytic performance through structural disruptions. This process involves intentional modifications to the nanomaterials’ structure to improve their ability to absorb light, generate charge carriers, and increase the efficiency of photoreactions on their surfaces. The primary strategies in defect engineering include the creation of vacancy defects, grafting of functional groups to modify the band structure, and improving crystallinity to extend the conjugation of the π system while weakening interlayer van der Waals interactions [79]. Particularly, vacancy defects—such as internal induced vacancies (Figure 6a,b), external induced vacancies (Figure 6c,d), cation (Figure 6e), and anion vacancies (Figure 6f)—can assist in regulating optical absorption, charge isolation, and surface photoreaction, resulting in increased photocatalytic efficiency [80]. Among these defects, oxygen vacancies, a form of anion vacancy, have been highly researched because they possess a low formation energy and can narrow the bandgap through external donor sites [81]. Oxygen vacancies can be deliberately created in semiconductor oxide photocatalysts by removing oxygen from the lattice under oxygen-deficient conditions [80]. The lower energy required for oxygen vacancy defects, as well as the defective electronic states emerging above the valence band, can be effectively used to balance the bandgap so that a broader wavelength of visible light can be absorbed. In addition, owing to its high electronegativity, the oxygen atom forms positively charged vacancies that facilitate photocarrier separation [82].

#### 2.3.3. Surface Modification and Morphology Control

The efficacy of photocatalytic processes is intrinsically linked to the physical characteristics of the catalysts, including their morphology, size, and crystal facets. These factors determine the surface area, the number of exposed active sites, and the extent of contact between the catalyst and reactants, thereby influencing adsorption properties. Specifically, the variation in morphology affects the specific surface area and the accessibility of active sites, which are crucial for the adsorption of reactants and the subsequent photocatalytic reactions. Moreover, the size of semiconductor photocatalysts plays a dual role by not only affecting their specific surface area but also inducing quantum size effects at the nanoscale. These effects lead to a broadening of the bandgap, which enhances the oxidative and reductive abilities of the catalysts. Additionally, the efficiency of charge separation varies across different crystal facets, with the arrangement of atoms, electronic structure, and presence of defects on each facet influencing the adsorption capacity and selectivity towards reactant molecules.

**Figure 7:**
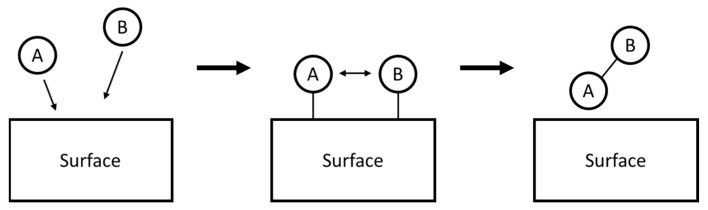
Illustration of the Langmuir-Hinshelwood Mechanism: Molecules A and B absorb onto the surface and interact to form a product, which then desorbs.

The Langmuir–Hinshelwood mechanism (Figure 7) elucidates that an increase in the specific surface area of a photocatalyst amplifies its photocatalytic activity [89,90]. This mechanism suggests that the photocatalytic reaction unfolds through an activated complex on the surface, following the adsorption of two reacting molecules on adjacent sites [91]. Given that the adsorption and desorption rates surpass the surface reaction rate, the latter becomes the rate-limiting step. Consequently, a larger surface area not only provides more active sites but also facilitates greater adsorption of target pollutants, thus enhancing photocatalytic efficiency.

When photoexcited, photocatalysts transport charge carriers to the surface, thereby instigating chemical reactions in the adsorbed molecules. An optimal photocatalyst ensures minimal recombination of these electron-hole pairs while maximizing electron and hole conveyance to the adsorbate, thus enabling efficient photo-reduction and oxidation reactions [92]. As such, the interfacial attributes of the photocatalysts become paramount, playing a pivotal role in bolstering charge separation and transfer dynamics within photocatalytic reactions. Jiang et al. [93] reported the successful synthesis of hydroxy-rich ultrathin BiOCl via a simple room-temperature method. These abundant surface hydroxyl groups in BiOCl-X contribute to improved dispersion in water, increase surface-active sites, and accelerate charge transfer. Consequently, BiOCl-X exhibits a significantly enhanced photocatalytic ability for degrading MPs, with a mass loss 24 times higher than that of BiOCl nanosheets. Furthermore, the study found that smaller MPs degrade more rapidly, while the light color or stable properties of MPs considerably reduce the photocatalytic degradation effect. Acidic conditions facilitate MP degradation, whereas alkaline conditions and Coulomb repulsion offer protection against degradation. Electron paramagnetic resonance and capture experiments revealed that surface hydroxyl groups in BiOCl effectively boost hydroxyl radical production, playing a crucial role in MP degradation. These findings suggest that the photocatalytic degradation of MPs relies on the surface hydroxyl groups of BiOCl, and a deeper understanding of reactive oxygen species in surface hydroxy-rich photocatalysts may contribute to more effective MP pollution control. 

#### 2.3.4. Nobel Metal Deposition (Plasmonic Photocatalysts)

**Figure 8:**
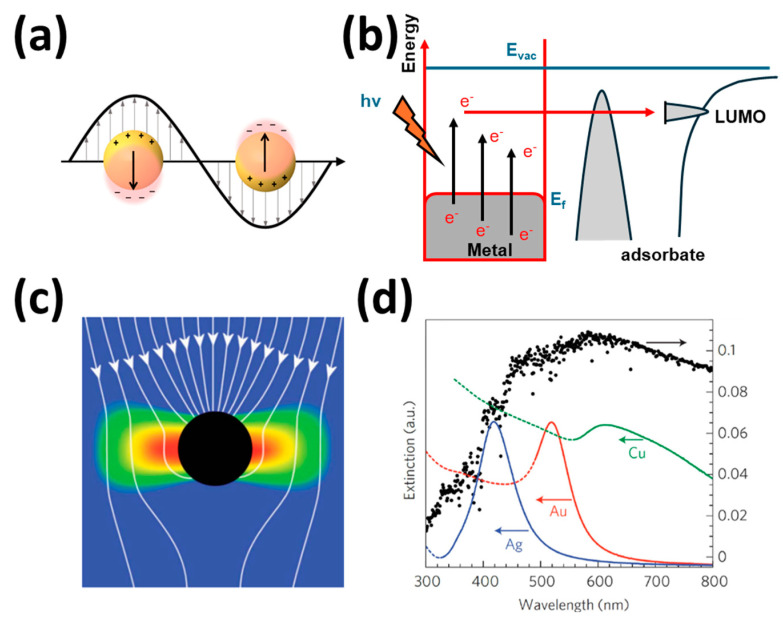
Plasmon-based photocatalysts: (**a**) Localized SPR plasmon resonance electric field on a sphere where the arrow shows the polarized electron movement. Reprinted with permission from [98], Copyright 2017, American Chemical Society. (**b**) Pure metal plasmonic photocatalysis [99]. (**c**) Energy flux (Poynting vectors) shown passing through plasmonic particles, where the red/blue is high/low electric field intensity. Reprinted with permission from [100], Copyright 2011, Royal Society of Chemistry. (**d**) Normalized extinction spectra of spherical Ag, Au, and Cu nanoparticles. Reprinted with permission from [101], Copyright 2016, Elsevier Inc.

To solve the weak light absorption problem associated with most semiconductors, plasmonic photocatalysis with localized surface plasmon resonance (LSPR) has emerged as a promising technology in recent years [94,95]. By establishing a Schottky barrier at the interface, dispersed noble metal NPs such as gold or silver NPs are capable of absorbing visible or even near-infrared wavelengths regardless of how much UV light traditional semiconductors have absorbed [96]. In response to the electromagnetic field of incident light, these noble NPs create an LSPR-driven collective oscillation of electrons (Figure 8a), which excites more electrons or holes through energy transfer or charge carrier transfer [97,98]. 

Pure plasmonic metal nanocrystals can also function as catalysts for chemical reactions. The excitation of LSPRs under light irradiation enhances catalytic reaction rates through two main mechanisms: plasmonic heating and hot electron transfer. Plasmonic heating increases the surface temperature via photothermal conversion. In hot electron transfer, excited electrons from plasmon excitation are injected into the lowest unoccupied molecular orbital (LUMO) of adsorbates, forming transient anions and weakening chemical bonds, thereby facilitating chemical transformations (Figure 8b) [99]. Hot holes can also interact with occupied molecular orbitals, enabling further reactions. Figure 8c illustrates the electric field intensity distribution and energy flux (Poynting vectors) around a plasmonic metal nanoparticle under incident electromagnetic radiation. This highlights how the nanoparticle absorbs photons from a larger area than its geometric cross-section, which is crucial for understanding and designing devices based on plasmon resonance energy transfer (PRET) [100].

As shown in Figure 8d, the extinction spectra of Au, Ag, and Cu nanoparticles indicate their activation by the SPR effect under visible light. This suggests that incorporating noble metal nanoparticles could significantly enhance the visible-light-driven photocatalytic performance of bismuth-based compounds [101]. For example, a recent study by Maulana et al. [102] demonstrated the decrease in the bandgap of TiO_2_ due to the presence of silver NPs, which was thus able to fully degrade polyethylene (PE) at an initial concentration of 100 ppm within 120 min. In addition, by using photo-assisted deposition (PAD) and reduced graphene oxide (rGO) via the ultrasonic radiation method, Fadli et al. [103] synthesized Ag/TiO_2_/rGO two-dimensional photocatalysts to degrade PE under UV radiation, and the results showed that the two-dimensional photocatalysts outperformed pure TiO_2_ and Ag/TiO_2_ in the degradation of PE.

The plasmonic effects depend on various factors, such as free carrier concentrations, morphologies, particle sizes, and distances [104,105]. According to desired properties and intended application, metal-based plasmonic materials can be readily synthesized using a range of techniques, such as hydrothermal, chemical vapor deposition (CVD), physical vapor deposition (PVD), and biogenic synthesis [106,107]. It is worth noting that the distinction between plasmonic photocatalysts and metal-doped photocatalysts is not mutually exclusive. Any composite material incorporating noble metal nanoparticles (NPs) that exhibit plasmonic effects can be classified as a plasmonic photocatalyst.

### 2.4. Photocatalysts with Support Materials

Addressing the intricacies and economic challenges associated with large-scale NP recovery [108] necessitates innovative approaches. One prevailing strategy involves the deployment of supporting materials to bolster photocatalyst efficiency without compromising recyclability. For example, Alle et al. [109] employed open-cell β-silicon carbide alveolar foams as the support material for TiO_2_ (P25) NPs, which was able to degrade 50% of the carbon of polymethylmethacrylate (PMMA) nanobeads in 7 h under UV-A light irradiation. Properly selected supporting materials can serve the dual purpose of enhancing photocatalysis efficiency while ensuring reusability.

To further enhance the effectiveness of photocatalysts, Zhang et al. [110] developed a novel solid-phase photodegradation of MPs and demonstrated complete mineralization of PS MPs using 30 nm TiO_2_ nanoparticle photocatalysts fabricated with Triton X-100 as a nonionic surfactant. Comprehensive mechanistic studies, including mass spectrometry and in situ diffuse reflectance infrared Fourier transform spectroscopy (DRIFTS), revealed that hydrocarbon ion fragments generated during polystyrene (PS) degradation were eventually transformed in CO_2_. With a degradation efficiency of 98.4%, this method represents the highest efficiency among photocatalysts reported to date. 

In a synergistic blend of environmental remediation and resource production, some photocatalysts have demonstrated dual functionalities. For instance, Meng et al. [111] reported a biomass-derived 3D MoS_2_/RGO/cotton photocatalyst capable of degrading PE and producing solar-driven freshwater. After 60 min of solar irradiation, a 12% weight loss of PE was observed, with large cavities and cracks on the PE surface indicating decomposition by reactive oxygen species.

### 2.5. Factors Influencing Photocatalytic Degradation of Microplastics

Microplastics (MPs) exhibit a diverse array of physical and chemical properties due to their varying sizes, compositions, morphologies, additives, contaminants, aging states, and surface charges [112,113]. This diversity introduces significant complexity to their photocatalytic degradation. Existing studies have shown some effectiveness in degrading certain types of MPs, particularly those with lower strength, hardness, and rigidity, such as polyethylene (PE). However, the success of the photodegradation process depends heavily on a multitude of parameters. Table 2 summarizes the usage of various photocatalysts to degrade different types of MPs from previous studies and clearly illustrates that there are significant differences between the operating parameters among those studies. Factors such as pH [114], temperature [60], the type and size of both MPs and photocatalysts [115], duration of radiation exposure [116], and light source [117] all substantially affect the photodegradation process. A study conducted by Llorente-García et al. [115] also exemplifies the intricacies of the process from another perspective, indicating that the degradation of PE is determined by several interrelated factors within the reaction system. These factors include (1) the abundance of hydroxyl radicals (OH•) generated from the high surface area of the coating, (2) the interaction between bulk and surface-bound OH• radicals and MPs (MPs) facilitated by diffusion and stirring, (3) the enhanced surface-to-volume ratio of smaller MPs, and (4) the shape of the MPs, which could create either good or poorly illuminated and oxygenated reaction environments. Furthermore, despite extensive research, an affordable and effective method for MPs elimination remains elusive. The variability of the photodegradation process, which can last from a few days to several weeks depending on the aforementioned factors, further complicates its large-scale application. Therefore, a detailed cost-benefit analysis, evaluating treatment costs, catalyst reusability, and potential ecological impact, is essential for realizing large-scale implementation of photocatalytic technologies. An interesting study by Liu et al. [118], for instance, reported that PS was even able to enhance the photodegradation of PP due to the photosensitization nature of PS and its released dissolved organic matter. These findings emphasized that considering and fine-tuning these factors is vital for MP degradation. However, these factors are not standardized, adding another layer of complexity to the process. Thus, these variables underscore the pressing need for continuous research to refine photocatalytic systems.

## 3. Photocatalytic Mechanisms in Nanomaterial-Mediated Plastic Degradation

Photocatalysis can be categorized into two types: photodegradation and photosynthesis [16]. Photodegradation involves the non-selective oxidation of plastics into smaller fragments, which can range from MPs and nanoplastics to small molecules and, ultimately, to CO_2_ [124]. This degradation process typically occurs with air or oxygen in the reaction system [125]. The process is facilitated by photogenerated holes and electrons, which create radicals such as hydroxyl radicals, driving the oxidation and breakdown of plastic materials. On the other hand, photosynthesis is an upcycling process that reduces carbon- and hydrogen-rich plastics and selectively produces recoverable and value-added target products, including fuels, chemicals, and materials [16]. However, photosynthesis is not efficient, and it can be difficult to recover small, dispersed MPs in the environment. Therefore, we are mainly focused on the photodegradation of MPs in this review.

Photocatalysis processes include (1) photon absorption and electron-hole pair excitation, (2) photogenerated charge carrier separation and migration, and (3) surface catalytic chemical redox reactions [16,126]. Each of these steps plays a crucial role in the efficiency of photocatalytic systems. First, effective light harvesting ensures that the materials absorb a sufficient amount of photons, leading to efficient electron–hole pair generation [127]. Second, charge separation and transfer processes are vital to prevent the recombination of these electron-hole pairs, enabling them to be transferred to the desired reaction spot [128]. Lastly, surface catalytic reactions are responsible for the conversion of reactants into products, ultimately determining the overall performance of the photocatalytic system. Optimizing each of these processes is crucial for advancing photocatalysis efficiency since they work synergistically to deliver the desired photocatalytic reaction. 

### 3.1. Techniques Used to Investigate Mechanisms

The plastic weight loss method is typically used to evaluate photodegradation efficiency, with CO_2_ being recognized as the final degradation product. This method, however, may not accurately reflect the process’s true efficiency, particularly for smaller fragments, and cannot be used in photosynthesis. Thus, more sophisticated characterization techniques may be required to gauge the extent of degradation and intermediate product formation. Using a combination of methods allows researchers to gain a more detailed understanding of the photodegradation process. 

**Figure 9:**
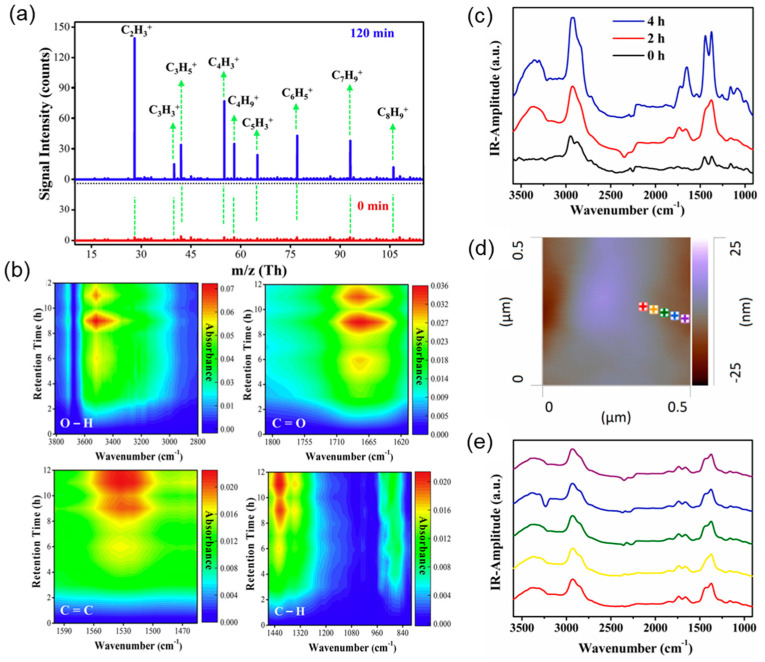
(**a**) Mass spectra obtained by high-pressure photon ionization (HPPI)–TOFMS during the photodegradation of PS. (**b**) DRIFTS study of PS at different time intervals. Reprinted with permission from [110]. © 2020 Elsevier Inc. (**c**) Localized nanoscale AFM–IR spectra of nano–TiO_2_ coated polypropylene microplastics after photo–aging for different time periods; (**d**) topographical images of TiO2 coated MPs after photocatalytic aging for 2 h; (**e**) AFM-IR spectra at different positions on the surface of MPs shown in Figure 9d. Reprinted with permission from [130]. © 2020 Published by Elsevier B.V.

Numerous existing techniques can be employed for the characterization of photocatalytic MPs to examine surface morphological changes, the formation of functional groups, free radicals, and other by-products. For example, Cho et al. [129] investigated the photodegradation of the poly(vinyl chloride) (PVC) films w/wo TiO_2_ using weight loss monitoring, scanning electron microscopic (SEM) analysis, gel permeation chromatography (GPC), FT-IR and UV–VIS spectroscopy, and X-ray photoelectron spectroscopy (XPS). The results showed a consistent reduction in the average molecular weight (Mw) of the composite film over time with exposure to light. By the end of 300 h of irradiation, the majority of the higher molecular weight fraction, which initially eluted between 13–15 min, had dissipated, reducing the average Mw to one-third of its initial value. Nabi and co-workers [110] used DRIFTS and high-pressure photon ionization (HPPI) (Figure 9a) coupled with time-of-flight mass spectrometry (TOFMS) (Figure 9b) to detect the formation of various hydrocarbon ions and functional groups during the photodegradation of polystyrene (PS), such as C_2_H_3_^+^, C_3_H_3_^+^, C_3_H_5_^+^, C_4_H_3_^+^, C_5_H_3_^+^, C_6_H_5_^+^, C_7_H_9_^+^, and C_8_H_9_^+^, which eventually transformed into CO_2_. In addition, Luo et al. [130] utilized an atomic force microscopy-infrared (AFM-IR) (Figure 9c,d) instrument to investigate surface coatings of MPs. He and his colleagues also detected an increased signal intensity of vinylidene end groups and a gradual decrease in the average softening temperature of MPs through FTIR spectroscopy and localized thermal analysis (TA) as irradiation time increased. Furthermore, the mechanical properties of MPs were able to be analyzed by Lorentz Contact Resonance (LCR) measurements of a contact resonance AFM. Furthermore, ^14^C isotope tracer, spectroscopic, and chromatographic techniques were also used by Tian et al. [131] to investigate the photodegradation process of PS NPs in air and water. Researchers can utilize these advanced analytical methods to gain a deeper understanding of photocatalytic processes, shed light on underlying mechanisms, and optimize material properties. 

### 3.2. Mechanism of Photocatalysis

Generally, there are two principal mechanisms that govern photocatalytic processes, i.e., the free radical mechanism and the singlet oxygen mechanism. The underlying pathways of these processes play an essential role in determining the efficacy of degradation and will be discussed in greater detail in the following sections.

#### 3.2.1. Free Radical Mechanism

After absorbing light, the photocatalysts generate and separate photoelectrons in the conduction band and holes in the valence band [132]. The generated photoelectrons and holes will migrate to the surface of the photocatalyst and react with other chemical species [133]. Specifically, a photoelectron that reaches the catalyst surface and encounters O_2_ molecules facilitates its reduction to O_2_•^−^ radicals, which subsequently react with water molecules and produce unstable •OOH radicals. In the next step, the radicals may break down into hydrogen peroxide and oxygen molecules and, under further irradiation, hydrogen peroxide can further break down into radicals known as •OH radicals. The highly reactive •OH radicals are the key oxidizing agent in most of the advanced oxidation processes and are capable of degrading plastics [134,135,136].

(1) $$semiconductor\overset{h\nu }{\to }h_{VB}^{+}+e_{CB}^{-}$$

(2) $$O_{2+}e_{CB}^{-}\to O_{2}^{•-}$$

(3) $$O_{2}^{•-}+H_{2}O\to \cdot OOH+{OH}^{-}$$

(4) $$2\cdot OOH\to O_{2}+H_{2}O_{2}$$

(5) $$H_{2}O_{2}\overset{h\nu }{\to }2\cdot OH$$

Meanwhile, the holes (h^+^) located in the valence band can also migrate to the photocatalyst surface, where they oxidize plastics directly or react with H_2_O adsorbed to generate more •OH, which in turn degrades the plastic into a variety of small molecular organic compounds or CO_2_ and H_2_O [16].

(6) $$h_{VB}^{+}+H_{2}O\to \cdot OH+H^{+}$$

A normalized decomposed process is suggested below. To be more specific, the weak spots (such as chromophoric groups or defects) of MP will first be initiated by the generated reactive oxygen species (•OH) and alkyl radicals [29]. Then, the oxygen will be incorporated into oxygen molecules and thus can easily lead to chain cleavage, branching, and cross-linking.

(7) $$-(CH_{2}CHR)-+\cdot OH\to -(CH_{2}C\cdot R)-+H_{2}O$$

(8) $$-(CH_{2}CHR)-+\cdot OH\to -(\cdot CHCHR)-+H_{2}O$$

(9) $$-(CH_{2}C\cdot R)-+O_{2}\to -(CHC(OO)\cdot R)-+H_{2}O$$

(10) $$-(\cdot CHCHR)-+O_{2}\to -(C(\cdot OO)HCHR)-+H_{2}$$

(11) $$-(CHC(OO)\cdot R)-+-(CH_{2}CHR)-\to -(CHC(OOH)R)-+-(CH_{2}C\cdot R)-$$

(12) $$-(C(\cdot OO)HCHR)-+-(CH_{2}CHR)-\to -(C(OOH)CHR)-+-(\cdot CHCHR)-$$

(13) $$-(CHC(OOH)R)-\overset{h\nu }{\to }-(CH_{2}C\cdot OR)-+\cdot OH$$

(14) $$-(C(OOH)CHR)-\overset{h\nu }{\to }-(CH\cdot OCHR)-+\cdot OH$$

The carbonyl radicals generated can further decompose through the Norrish reactions, which are a series of photodegradation processes that involve the cleavage of polymer chains under irradiation [137]. The Norrish reactions were categorized based on their mechanistic pathways, radical or non-radical nature, and the types of chemical species produced as a result of the reactions [138]. The Norrish type I reaction entails the cleavage of the bond between the carbonyl group and the α-carbon atom (α-scission), leading to the formation of carbon (II) oxide (CO) [137]. The Norrish type II reaction is a non-radical process that involves hydrogen atom abstraction from the γ-carbon atom, resulting in the decomposition of the polymer into an unsaturated chain and a chain with a carbonyl group end [139]. At the same time, the Norrish type III reaction is another non-radical chain scission process that involves the transfer of a β-hydrogen atom, ultimately yielding an olefin and an aldehyde [29,137,140,141]. The likelihood of each Norrish reaction occurring varies depending on their respective activation energies, which dictate the ease of the reaction to take place under specific conditions [137,141]. 



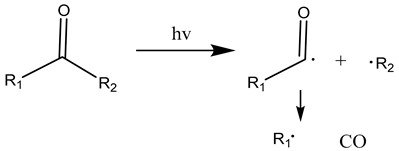

(15)




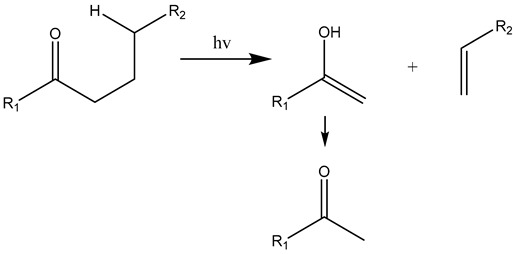

(16)




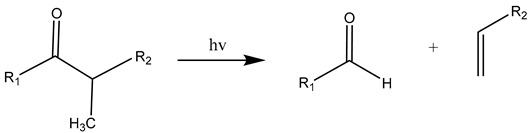

(17)


Then, polymers become more brittle, and the molecule’s weight decreases as a result of the effect of the entire process, which further facilitates degradation. Lastly, upon further oxidation, mineralization occurs to produce carbon dioxide and water, as explained below. For example, Meng et al. [111] utilized a biomass-derived three-dimensional MoS2/RGO/cotton photocatalyst for the photodegradation of PE, achieving mineralization to CO_2_ through C–C bond cleavage under attack by reactive oxygen species such as •OH and O_2_•^−^. The complete mineralization process was corroborated by the loss of PE weight, reflecting the high mineralization efficiency of this system. This elucidates the specificity of the photocatalytic degradation mechanism, where even complex polymer structures are broken down into carbon dioxide and water.

(18) $$-\left(CH_{2}C\cdot OR\right)-\left(or-\left(CH\cdot OCHR\right)-\right)+O_{2}\overset{photocatalysts,h\nu }{\to }intermediates\;such\;as\;RCH_{2}CHO,RCHO,and\;RCOOH$$

(19) $$-\left(CH_{2}CHR\right)-,RCH_{2}CHO,RCHO,and\;RCOOH\overset{photocatalysts,h\nu }{\to }CO_{2}+H_{2}O$$

#### 3.2.2. Singlet Oxygen Oxidation Mechanism

An alternative mechanism to photodegrade MPs is through oxidizing singlet oxygen, which can be generated through photochemical, thermal, chemical, and enzymatic reactions [142,143]. In this mechanism, singlet oxygen—a highly reactive form of molecular oxygen—directly reacts with and oxidizes plastics, causing subsequent chain scission [144,145]. The generation of singlet oxygen relies on the presence of suitable sensitizers, which are molecules capable of absorbing light and transitioning to a triplet state [146]. This triplet state can then facilitate the conversion of triplet-state oxygen into the highly reactive singlet state [147]. By harnessing the reactive power of singlet oxygen, this mechanism provides a promising avenue for the photodegradation of plastics through direct oxidation.

(20) S3+O23→S1+O21

**Figure 10:**
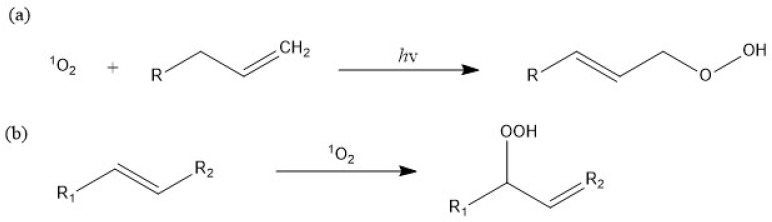
Singlet oxygen mechanism of oxidation. (**a**) Singlet oxygen oxidation of a vinyl group to the hydroperoxide functional group (ROOH) [150]; (**b**) singlet oxygen oxidation of an olefin group to the hydroperoxide functional group [149].

The singlet oxygen formed is also capable of reacting with the vinyl group produced by a Norrish reaction [142]. This interaction prompts further decomposition of the molecule, culminating in chain scission and the emergence of the hydroperoxides functional group (ROOH), as illustrated in Figure 10. The detection of singlet oxygen can be achieved by EPR measurements, and the singlet oxygen is demonstrated to be able to promote selective styrene oxidation through Scavenger-assisted photocatalytic experiments, according to Cheng et al. [148,149].

## 4. Conclusions and Perspectives

The increasing prevalence of microplastics in our ecosystems has given rise to significant environmental and health concerns. Photocatalysis, as an eco-friendly solution, has garnered considerable attention for its potential in addressing different environmental problems. Notably, while numerous factors can influence the degradation process, the overarching determinant lies in the potency of the photocatalysts themselves. Through various material enhancement strategies, such as doping and heterojunction construction, the efficiency of this degradation process can be amplified. Moreover, a deep understanding of the underlying mechanisms, including the free radical mechanism and the singlet oxygen oxidation pathway, was introduced in this review and is essential for further optimization of the photodegradation efficiency. 

The exploration of those advanced modification strategies for photocatalytic nanomaterials in microplastic degradation remains limited, such as defect-engineering and surface and morphology modification, signifying a potent area for groundbreaking progress in environmental remediation. Except for the photocatalysts modification, future directions of the photodegradation should address the sustainability of photocatalytic processes, emphasizing the long-term stability, reusability, and economic feasibility of these nanomaterials. It is also paramount to evaluate the environmental implications of degradation products and any potential toxicity associated with the photocatalysts themselves. 

The economic viability of photocatalytic processes for handling plastic waste is critical for their large-scale implementation. While photocatalysis offers a potentially low-cost solution by using sunlight as an energy source, the initial investment in photocatalyst materials and system integration, along with ongoing maintenance costs, must be considered. Advances in material science have led to more efficient photocatalysts, but the high-performance materials currently available, such as titanium dioxide and zinc oxide, still pose significant economic hurdles.

For photocatalytic processes to achieve commercial scalability, addressing the challenge of large-scale synthesis of photocatalysts with consistent quality and performance is essential. The design and engineering of reactors capable of handling substantial volumes of plastic waste, ensuring adequate light penetration and photocatalyst distribution, are also crucial for scalability. Innovations in reactor design, such as photoreactors with enhanced light-harvesting capabilities, are necessary to maintain efficiency at larger scales. The effectiveness of photo-oxidation is further constrained by variable environmental conditions, such as light availability, particularly in marine environments where conditions can severely limit its potential. Therefore, photo-oxidation should be integrated as one component of a broader strategy that includes improving waste management practices, developing biodegradable alternatives, and deploying targeted photo-oxidation technologies in conducive environments, ensuring a comprehensive and practical approach to tackling microplastic pollution.

Despite the promising aspects of photocatalytic processes, several challenges remain for their widespread adoption in plastic waste management. Photocatalyst deactivation over time due to fouling or structural changes requires frequent replacement or regeneration. The complexity and diversity of plastic waste, containing various additives and contaminants, can hinder the catalytic process. In addition to advancing photodegradation techniques, it is essential to protect our environment by enhancing recycling and upcycling processes for larger plastic items, preventing them from breaking down into microplastics and further polluting our ecosystems. Moreover, developing appropriate methods for the separation and enrichment of degraded products is crucial to addressing the issue of environmental microplastics more effectively.

In summary, while photocatalytic processes for plastic waste handling present a promising sustainable solution, their economic viability and commercial scalability are contingent upon overcoming significant material, engineering, and operational challenges. Ongoing research and technological advancements are essential to address these issues, paving the way for the successful integration of photocatalytic technologies into broader waste management infrastructure. This holistic view, encompassing environmental, technical, and economic facets, is necessary as the challenge posed by microplastics necessitates ongoing innovations and collaborations.

## Figures and Tables

**Table 1:** Comparisons of microplastics removal techniques.

Method	Efficiency (%)	Advantages	Drawbacks	Targeted MPs	Size	References
Adsorption on Green Microalgae	94.5 (recovery rate)	High affinity, selective based on surface charge	Nonrecyclable, chemical adherence, surface poisoning	PP	>400 μm	[30]
Dynamic Membranes	>80 (NTU)	Low resistance, easy operation, nonchemical treatment	Frequent cleaning, energy demand, sludge accumulation	N/A	1.65–516 μm (>90 μm)	[31]
Advanced Membrane Bioreactor	Usually > 98%	Combined with other advanced treatment	Shape dependency, membrane fouling	fibers (82%) and particles (18%)	mostly < 1 mm	[32]
Coagulation and Agglomeration	91.45	Removes small microparticles, controllable conditions, simple devices, low cost	Chemical addition, not suitable for large MPs	PET	<500 μm	[33]
	97–98.3			PE/PP	318 ± 258 μm	[34]
Hydrolysis	Complete degradation	Fast and cheap	High energy demand, strongly alkaline	Partially degraded polyester MP fibers	20–25 μm	[22]
Ozonation	44	no chemicals added, effective than chlorination, irrelated to pH	Low solubility, higher expenses than chlorination, leave residuals and byproducts	PE	Various sizes	[35]
Chlorination	7.1	Cheap, and chlorine residuals still work	Poor treatment effect, leaves a chlorinated taste, may cause skin irritation	nano-sized PS	<1 μm–5 mm	[36]
UV-induced Oxidation	N/A	No chemicals needed	Require extreme dose irradiation, low efficiency	PS, PE, PVC, PET	150–300 nm	[37]
Biological Ingestion	66.03	Simple, low operating costs, wide applicability, flexible	uncontrollable environmental conditions, difficulty analyzing products, reproducibility	PE	53–500 μm	[38]

N/A: Not applicable.

**Table 2:** Reaction conditions of selected MPs with different MPs.

MP Type	Size	Photocatalyst (w or w/o Carrier)	Size	Irradiation	pH	Temperature (°C)	Exposure Time (h)	Results (%)	Reference
PE	700–1000 µm	MP/N-TiO_2_ film composite	220 to 920 nm	27 W fluorescent lamp (400–800 nm)	-	room temperature	18	0.064	[119]
	725 ± 108 µm	C,N-TiO_2_ particles	-	50 W LED light (400–800 nm)	3	0	50	0.7177	[60]
	382 µm	N-TiO_2_ on Pluronic^®^ F127 membrane	12 ± 3 nm	50 W Visible LED Lamp (400–800 nm)	3	-	50	0.0465	[115]
	200–250 µm	hydroxy-rich ultrathin BiOCl	10–40 nm	250 W Xe lamp with UV cut-off filter (λ > 420 nm)		-	10	5.38	[93]
PS	400-nm	TiO_2_ on Triton X-100 membrane	-	UV	-	room temperature	12	98.4	[110]
	3 µm	Au@Ni@TiO_2_ micromotors	700 nm + 10 nm Ni + 30 nm Au	UV	-	-	-	71	[120]
PP	154.8 ± 1.4 µm	ZnO nanorods	~1.6 µm long and ~200 nm wide	visible light	-	-	456	65	[121]
PMMA	105 nm	β-SiC foam-supported TiO_2_–P25	-	UVA (354 nm)	6.3	-	7	50	[109]
Polyamide 66 fiber	10 μm in diameter, 1.0 mm in length	TiO_2_ P25 powder	21 nm	UVC (254 nm)	-	25–38	48	97	[122]
PET fiber	~25 μm in diameter, 5 mm in length	Pt@N-TiO_2_-1.5%	500 nm	300 W Xe lamp with AM 1.5 filter	-	-	48	29	[62]
PVC	-	Nb_2_O_5_ atomic layers	-	300 W Xe lamp with AM 1.5 G filter	-	~25	90	90	[123]
